# Do the unemployed hit the bottle during economic downturns? An empirical approach for Spain

**DOI:** 10.1186/s12889-019-6882-2

**Published:** 2019-05-07

**Authors:** Carla Blázquez-Fernández, David Cantarero-Prieto, Patricio Perez

**Affiliations:** 1 Department of Economics, Universidad de Cantabria, Av. Los Castross/n, 39005 Santander, CP Spain; 2 Instituto de Investigación Sanitaria Valdecilla. IDIVAL, Santander, Spain; 3 GEN Governance and Economics Network, Universidad de Vigo, Vigo, Spain

**Keywords:** Spain, Alcohol consumption, Crisis, Unemployed, Discrete-choice models, Matching techniques

## Abstract

**Background:**

This paper analyses the 2008 economic collapse in Spain with its long-lasting effects. Precisely, the ones associated with lifestyles. Thus, the aim of this paper is to examine to what extent economic downturns affect individual’s drinking behavior when focusing on unemployed people.

**Methods:**

We use discrete-choice models and matching techniques. Data from the National Health Survey for 2006 and 2011–2012 provides a clear picture before and after the 2008 breakdown in Spain.

**Results:**

We find that drinking over the business cycle is a function of individual socio-demographic status. Besides, our empirical findings are consistent with the idea that following the crisis differences between unemployed and non-unemployed fell to at least in accordance with a lower overall consumption of alcoholic beverages.

**Conclusions:**

Public policy design for drinkers would require both prevention and recovery from alcohol use strategies to be met towards health and labour pillars.

**Electronic supplementary material:**

The online version of this article (10.1186/s12889-019-6882-2) contains supplementary material, which is available to authorized users.

## Key points

- The study contains evidence on unlike drinking behavior over the business cycle.
- The episode analyzed is the 2008 economic collapse in Spain.
- Public policy design would require both prevention and recovery strategies.

## Background

While it is generally acknowledged that long-term economic growth enhances health, the debate over the impact of short-term economic shifts remains open [1, 2]. Basic intuition suggests that health should somewhat improve through expansions and decline in recessions [3]. However, findings from previous papers are surprisingly mixed [4–8]. Additional studies identifying the contributory mechanisms through which changes in economic activity may affect health are needed.

This paper contributes to the recent literature on the associations between economic crises and alcohol drinking behavior when considering Spain as a particular scenario [9]. Two facts are important in this regard. First, since the financial crisis of 2008 Spain experienced an increase in joblessness that is above average in relation to the one within the European Union. Accordingly with Eurostat, this rate rose from 8.2% in 2007 to 26.1% in 2013 whereas for the European Union-28 it takes 7.2 and 10.9%, respectively. As a result, Spain registered a greater rise in employment inequality than any other OECD country during period 2007–2011 [10]. Second, the Mediterranean tradition of viticulture is so deeply rooted in Spain that alcohol drinking could become trivialized. To what extent might this crisis have contributed to reduced health capital and hence income growth?

The aim of this paper is to examine the association between employment status and drinking behavior over the business cycle in Spain. It should be noted that this study does not examine the effect of alcohol consumption on labor supply. At the macro-level, this paper assesses whether alcohol abuse is associated with economic downturns, and if so to what extent. At the individual-level, the more relevant underlying pathway is the one from labor status from subjects who are prone to alcohol misuse. For both levels, we jointly focus on the Great Recession which started in 2008 as reflected in data collected from the Spanish National Health Survey (SNHS) in 2006 (before the crisis) and 2011–2012 (throughout the crisis). Taking advantage of the modern economic crisis, our goal is to analyze whether drinking patterns are driven by this episode. We use a unified framework embedding discrete-choice models and matching techniques. In doing so, we are able to assess the robustness of our results to alternative specifications.

Several features distinguish this paper from previously published studies. First, this paper focuses on unemployment and alcohol consumption in Spain in terms of two opposite phases of the business cycle. Second, on the empirical side our paper highlights specific risks associated with economic conditions which provide crucial information when it comes to designing public-health policies. For the same reason, governments should be actively engaged and placing more emphasis on the impacts of health capital on the well-being of citizens.

## Methods

### Measuring alcohol consumption and specification

A demand model can be used to illustrate how economic downturns are associated with drinking behavior [11]. Algebraically, the demand for health *Y* for individual *i* is represented as follows:

1 $$ {Y}_i=\kern0.5em {F}_i\left(W,P,X,\varepsilon \right) $$

where *W* means wages, *P* represents prices, *X* denotes personal characteristics and *ε* indicates error term. It should be noted from Eq. (1) that wage is likely to be endogenous due to unobserved factors that affect labor market choices, and it is not fully available. In line with this view, employment status and time of unemployment are among a low number of determinants of demand for health (*X*), which include individual-specific characteristics.

The formulation of the problem can be illustrated within the framework of Mullahy [12] and Ruhm and Black [4]. In our model, the expected consumption by individual *i* is determined by:

2 $$ E\left[{Y}_i\left|{X}_i\right.\right]=\Pr \left[{Y}_i>0\left|{X}_i\right.\right]\times E\left[{Y}_i\left|{Y}_i>0,{X}_i\right.\right] $$

where *Y* is the number of drinks, *X* is some vector of individual-level characteristics, and *E*(·) and *Pr*(·) indicate conditional expectations and probabilities. The distinction between alcohol drinking participation and conditional alcohol drinking is standard in the health economics literature [13]. We observe *Y* as a result of comparing the utility of consuming a given (including zero) number of drinks with another number of drinks. The observability rule is *Y* = 1 (*Y*^∗^ > 0) for the binary choice of being a drinker or not being a drinker, or *Y* =  *max* (*Y*^∗^, 0) for the number of drinks consumed. Latent variable Y^*^ is a linear function of labor status (being unemployed) and additional characteristics (including age, gender, educational level, marital status, and nationality). The empirical counterpart to Eq. (2) is given by Eq. (3):

3 $$ \Pr \left({Y}_i=1\right)=\beta {U}_i+\kern0.5em {X}_{\mathrm{it}}{\Gamma}^{{}^{\backprime}}+{\varepsilon}_i $$

where *U* denotes unemployed, and *X* is a matrix of a constant term and the individual’s characteristics.

Next, we turn to study whether individuals have different drinking behavior due to the fact of being unemployed. To this end, we specify the average treatment effect on the treated (ATT). Following Ayala and Rodríguez [14] and more precisely Urbanos-Garrido and Lopez-Valcarcel [7], matching methods based on propensity score [15] are applied separately for the 2006 and 2011–2012 SNHS data (before and after the crisis). Those methods consider the effect in terms of potential outcomes or counterfactuals. Let the variable *D* be a binary treatment indicator, where *D* = 1 denotes treatment and *D* = 0 otherwise. The empirical model used here is:

4 $$ ATT=E\left[{Y}_1-{Y}_0\left|D=1\right.\right]=E\left[{Y}_1\left|D=1\right.\right]-E\left[{Y}_0\left|D=1\right.\right] $$

where subscripts 1 and 0 mean unemployed and non-unemployed, respectively, and *D* = 1 means unemployed. Matching techniques are based on comparing two groups: (i) individuals who have received treatment (treated group), and (ii) individuals who have not received treatment but have similar characteristics as those who received it (control group). Hence, the second term on the right-hand side of Eq. (4) is the counterfactual: what would the drinking behavior of an unemployed person be if he/she were employed?

Thus, we capture the effect of business cycle on drinking behavior through unemployment status as:

5 $$ {Y}_{idt}=\gamma {\mathrm{U}}_{\mathrm{it}}+\delta t+\uptheta \left({\mathrm{U}}_{\mathrm{it}\cdot}\mathrm{t}\right)+{X}_{it}{\Gamma}^{{}^{\backprime}}+{\varepsilon}_{idt} $$

where *d* stands for employment status, and *t* is a time dummy variable representing the period, during which the individual is observed (t = 1 if period is 2011–2012, or t = 0 if is 2006). It therefore collects all changes (e.g., prices, taxes or health expenditure) which occurred during this period and are not explained by the remaining explanatory variables. It should be noted from Eq. (5) that parameter *δ* represents the change in drinking behavior, whereas *γ* and *θ* capture the effects of unemployment status on drinking before (*γ*) and over the crisis (*γ* + θ). The advantage of this framework is that the economic crisis is safely assumed to be an exogenous shock, hence it is the dominant force for unemployment status. Therefore, looking at the effects on drinking behavior for 2006 avoids possible endogeneity biases from reverse causality [16].

The empirical strategy relies on two different approaches. First, estimates from Eq. (3) show the impact of being unemployed on the probability of drinking behavior when controlling for individual characteristics. In this regard, bivariate and multinomial models are used [17, 18]. Next, in order to get a better insight of the question, we extend the approach. From Eq. (4) we estimate the impact of being unemployed on alcohol consumption by using matching (and propensity score) techniques. Again, we calculate the probability of being unemployed (“treated”) as a function of the covariates *X* associated with unemployment for the two periods. The parameter of interest to be estimated is the average treatment effect on the treated (ATT) of unemployment on the unemployed. Having calculated the propensity score, we applied different methods for correlation-making purposes: nearest-neighbor, radius, and kernel. Briefly, nearest-neighbor matching involve matching individuals by the propensity score with the smaller difference. As for the radius method, each individual is matched with one of more individuals in the “control group” whose propensity score is closer than a prefixed given number. Regarding the kernel, the “treated” individuals are compared with a weighted average of the “control” ones, being the weights inversely proportional to the distance between the propensity scores of these two groups. Finally, from Eq. (5) we analyze the effect of the business cycle on drinking over unemployment.

## Data

Data for the empirical analysis come from the SNHS. It is a widely used research operation that is carried out by the Spanish National Institute of Statistics (INE). Comparable to other European health surveys, the objective is to obtain data from citizens regarding issues associated with health status and health-related behaviors. In this respect, two caveats need to be taken into account. First, the SNHS is not the typical periodical survey. We use the 2006 and 2011–2012 surveys because these are the available surveys for periods immediately before and after the Great Recession. By way of illustration, prior to 2006 only the 2003 survey was conducted, while survey data for the intervening years 2007–2010 are unavailable. Second, the 2011–2012 survey responders are not the same as the responders for 2006.

Samples consist of 31,300 and 24,000 dwellings, distributed among 2236 and 2000 census sections, for 2006 and 2011–2012, respectively. It should be noted that the percentage of the total effective sample (96.11%t for 2006 and 89.62% for 2011–2012) can be considered as the response rate in each survey. This response rate represents the percentage of households that has been included in the practice (including substitutions). Regarding non-response in each survey, it is derived by refusals, absences and inabilities to answer. In the 2006 survey it gets *n* = 8015 (28.39%) whereas it is *n* = 6004 (28.94%) in 2011–2012. Absences are those that have the greatest weight in the non-response in 2006 (15.98%) while the absences are in 2011–2012 (14.61%). The surveys include different items regarding alcohol consumption. Since the sale of alcohol in Spain is limited to persons aged 18 years and over, it implies that the scope of the study was reduced to include adult working-age people in the age range of 18–65 years (despite the fact that legal working-age in Spain is from 16 years of age). As a result, the sample provided for 2006 comes down to 14,696, while for 2011–2012 is 14,981.

We then outline by mean of operational definitions how to measure the consumption of someone drinking alcohol in order to define the subject’s score of dependent variables. Since questions concerning the subject vary somehow among surveys, these variables needs to be modified slightly. We are concerned about what impact this is likely to have on the study when trying to account for this by modifying the variables. It should be noted that we are limited by the variables included within the National Health Survey(s). That is to say, in both surveys respondents are asked whether they have consumed at least one drink of any alcohol beverage in the past two weeks, last year or a any previous time. Besides, other questions that are related with frequency of consumption. Differences between surveys are so, on how respondents are questioned but not at all in the final content of their answers that finally could be make uniform. Therefore, following Mossakowski [19], two proxies measuring characteristics related to “heavy” alcohol consumption are identified: *drinker* and *drinking-frequency.* For the former we created one dichotomous variable, coded 1 for those people consuming five or more drinks a week and 0 otherwise. In our sample 19.65% of the unemployed would be classified as drinkers in 2006, whereas this proportion has increased to 25.39% in 2011–2012. The second dependent variable measures the frequency of drinking without collecting the number of times; hence, it is not an ordered variable. This comprises four levels: 0 (does not drink alcohol), 1 (drinks alcohol only on weekends), 2 (drinks alcohol only on weekdays), and 3 (drinks alcohol on weekdays and weekends alike). The econometric model we use in dealing with *drinking-frequency* is a multinomial one. Drinking on weekends versus weekdays is not really ordered, although drinking on both weekdays and weekends or neither could be considered ordered.

Turning to the explanatory variables, we are interested in background demographic data which are common to all two surveys. *Unemployed* is a dichotomous variable for labor status: 1 if the individual declares being unemployed, 0 otherwise. *Gender*, *married,* and *nationality* are also dichotomous variables. Notwithstanding, foreigner seems potentially quite heterogeneous since there are not enough observations to disentangle category (race/ethnicity). *Age* is a continuous variable ranging from 18 to 65 years as stated above following previous contributions (e.g.: Mossakowski [19]) when the outcome of interest is a lifestyle factor versus when it is a health outcome. *Education* is categorized in terms of five levels of educational attainment, with “primary education or below” being the reference category. Personal income could not be considered in this study because it is not available for both surveys. Table 1 shows further details on the variables used in the estimates. Summary statistics for the samples are also provided.

**Table 1:** Definitions of Variables and Descriptive Statistics

Variables		Definition	2006 (*n* = 14,696)		2011–2012 (*n* = 14,981)	
			Mean	S.D.	Mean	S.D.
Drinker		1 if consumes five or more drinks a week	0.259	0.438	0.256	0.436
Drinking-frequency		0 if does not drink alcohol,				
		1 drinks only on weekends,				
		2 drinks only on weekdays, and	2.599	0.904	0.841	1.281
		3 drinks on weekends and weekdays alike				
Labour status	Unemployed	1 if unemployed	0.122	0.328	0.175	0.380
	Unem_never	1 if unemployed and has never worked	0.006	0.077	0.007	0.085
	Unem_6	1 if unemployed for 6 months or less	0.052	0.222	0.051	0.220
	Unem_6_12	1 if unemployed for 6 months to 1 year	0.017	0.129	0.028	0.165
	Unem_12	1 if unemployed for 1 year or longer	0.043	0.203	0.088	0.283
Age	Age	Age (in years)	40.495	10.813	43.319	12.557
Gender	Male	1 if male	0.477	0.499	0.489	0.500
Education	Educ1	Primary education or below (reference category)	0.289	0.453	0.139	0.346
	Educ2	Compulsory secondary education	0.136	0.343	0.342	0.474
	Educ3	Non-compulsory and pre-university secondary education	0.245	0.430	0.592	0.491
	Educ4	Specific labour training	0.094	0.292	0.078	0.269
	Educ5	University graduate	0.231	0.422	0.190	0.393
Marital status	Married	1 if married	0.583	0.493	0.551	0.497
Nationality	Nationality	1 if foreigner	0.096	0.294	0.102	0.303

## Results

We first focus on the impact of being an unemployed on *drinker* from Eq. (3). Table 2 reports the results according to the duration of unemployment both prior to and following the crisis. Estimates in column 1 of the 2006 survey indicate that considered as a whole the unemployed individuals are significant and negatively associated with alcohol abuse (0.789). Further, the results in columns 2–5 show that while the association is weak for the short-term unemployed, the association remains highly significant in the long-run (6 months or more) where coefficient gets 0.529–0.821. We obtain similar estimates in column 6 when introducing all the variables regarding the duration of unemployment (unemployed-never, understood as the unemployed has never worked, is not considered due to we focus on those that had worked whenever). These findings are consistent with the income hypothesis. Besides, we obtain evidence that education has no-significant coefficients on all but the highest level, which is negative (see i.e. coefficients associated with university graduate that stay at 0.85–0.86).

**Table 2:** Logistic Estimation. Dependent Variable: *drinker*

	(1)		(2)		(3)		(4)		(5)		(6)	
Variable/specification												
Pre-crisis (2006)												
Unemployed	0.789 [0.691–0.901]	***										
Unem_never			0.709 [0.358–1.404]									
Unem_6					0.886 [0.731–1.073]						0.868 [0.716–1.051]	
Unem_6_12							0.529 [0.362–0.774]	***			0.519 [0.355–0.760]	***
Unem_12									0.821 [0.666–1.011]	*	0.806 [0.654–0.993]	**
Age	1.037 [1.033–1.041]	***	1.037 [1.033–1.041]	***	1.037 [1.033–1.041]	***	1.037 [1.033–1.041]	***	1.037 [1.033–1.042]	***	1.037 [1.033–1.041]	***
Male	4.166 [3.834–4.526]	***	4.217 [3.833–4.581]	***	4.217 [3.883–4.580]	***	4.214 [3.880–4.577]	***	4.200 [3.887–4.563]	***	4.177 [3.845–4.538]	***
Educ2	0.983 [0.864–1.118]		0.989 [0.869–1.125]		0.988 [0.867–1.124]		0.989 [0.869–1.125]		0.987 [0.868–1.123]		0.984 [0.865–1.121]	
Educ3	0.898 [0.804–1.002]	**	0.909 [0.815–1.014]	*	0.908 [0.814–1.013]	*	0.907 [0.813–1.012]	*	0.906 [0.812–1.011]	*	0.900 [0.806–1.004]	*
Educ4	0.978 [0.842–1.136]		0.992 [0.855–1.151]		0.991 [0.853–1.150]		0.992 [0.855–1.151]		0.989 [0.852–1.147]		0.982 [0.846–1.140]	
Educ5	0.845 [0.755–0.946]	***	0.863 [0.771–0.965]	***	0.860 [0.769–0.961]	***	0.859 [0.768–0.960]	***	0.857 [0.767–0.959]	***	0.847 [0.757–0.948]	***
Married	0.958 [0.881–1.042]		0.971 [0.893–1.055]		0.969 [0.892–1.053]		0.965 [0.887–1.049]		0.967 [0.889–1.052]		0.957 [0.880–1.041]	
Nationality	0.650 [0.559–0.757]	***	0.651 [0.559–0.757]	***	0.652 [0.560–0.759]	***	0.650 [0.558–0.756]	***	0.649 [0.558–0.755]	***	0.649 [0.557–0.755]	***
Pseudo R^2^	0.106		0.105		0.105		0.106		0.105		0.106	
Variable												
Crisis (2011–2012)												
Unemployed	1.014 [0.914–1.126]											
Unem_never			0.587 [0.316–1.090]	*								
Unem_6					1.276 [1.074–1.514]	***					1.263 [1.063–1.501]	***
Unem_6_12							1.033 [0.816–1.309]				1.039 [0.820–1.318]	
Unem_12									0.884 [0.768–1.116]	*	0.899 [0.781–1.035]	
Age	1.027 [1.023–1.030]	***	1.026 [1.022–1.030]	***	1.027 [1.023–1.030]	***	1.026 [1.023–1.030]	***	1.026 [1.023–1.030]	***	1.027 [1.023–1.030]	***
Male	4.468 [4.112–4.855]	***	4.465 [4.109–4.850]	***	4.457 [4.102–4.843]	***	4.469 [4.114–4.856]	***	4.477 [4.121–4.865]	***	4.463 [4.108–4.850]	***
Educ2	0.909 [0.820–1.007]	*	0.912 [0.823–1.010]	*	0.903 [0.815–1.000]	**	0.909 [0.821–1.007]	*	0.914 [0.825–1.013]	*	0.906 [0.818–1.004]	*
Educ3	1.364 [1.191–1.561]	***	1.355 [1.184–1.550]	***	1.369 [1.197–1.567]	***	1.362 [1.190–1.559]	***	1.354 [1.183–1.550]	***	1.362 [1.190–1.560]	***
Educ4	1.352 [1.132–1.614]	***	1.342 [1.124–1.602]	***	1.359 [1.139–1.622]	***	1.350 [1.131–1.611]	***	1.338 [1.121–1.597]	***	1.348 [1.130–1.610]	***
Educ5	1.379 [1.196–1.590]	***	1.371 [1.189–1.580]	***	1.386 [1.202–1.597]	***	1.377 [1.195–1.587]	***	1.362 [1.181–1.570]	***	1.373 [1.191–1.584]	***
Married	0.825 [0.759–0.896]	***	0.823 [0.757–0.894]	***	0.825 [0.759–0.896]	***	0.825 [0.759–0.896]	***	0.821 [0.755–0.892]	***	0.822 [0.756–0.893]	***
Nationality	0.743 [0.645–0.856]	***	0.744 [0.647–0.857]	***	0.738 [0.641–0.850]	***	0.743 [0.646–0.856]	***	0.747 [0.649–0.861]	***	0.740 [0.643–0.853]	***
Pseudo R^2^	0.096		0.096		0.096		0.096		0.096		0.096	

95% CI in square brackets

*Education* is categorized in terms of five levels of educational attainment (*Educ1*-*Educ5*, with *Educ1* “primary education or below” being the reference category. *Educ2*: Compulsory secondary education; *Educ3*: Non-compulsory and pre-university secondary education; *Educ4*: Specific labour training; *Educ5*: University graduate

Observations: 14,696 for 2006 and 14,981 for 2011–2012

Reference category: the reverse one for each dichotomous variable

***p < 0.01, **p < 0.05, *p < 0.10

Conversely, we reject from column 1 of the 2011–2012 survey that being unemployed have significant impacts on *drinker* (“heavy” alcohol consumption). However, when time in unemployment is considered all variables are significant and positive, except long unemployment. It is consistent with both the provocation hypothesis and income hypothesis, attributing heavy alcohol drinking to stress caused by not being accustomed to being unemployed. Then, estimated coefficients on all but the lowest education levels show positive signs. But the crisis is significant not only in regard to being unemployed and education variables, being also significant concerning marital status. Therefore, the coefficient for being married is significantly negative in period 2011–2012, but not in 2006. In contrast, the estimates for age, gender (male), and nationality are robust to specification changes, the first two being positive, the coefficients associated with age are around 1.03 whereas male is about 4.56, and the third being negative, nationality gets 0.74, both before and during the crisis.

Next, we present evidence on the mechanisms driving *drinking-frequency*. Table 3 reports the results from Eq. (3), both for the 2006 and 2011–2012 surveys, when using not-drink-alcohol as the base outcome. Columns 1–3 present estimated effects for drinking on weekends, weekdays, and weekends-and-weekdays alike. It is noteworthy, that the estimates are as expected and they are consistent with the results from the full sample. Hence, when examining the influence of being unemployed on alcohol consumption, we again observed statistically negative impacts in the pre-crisis period (between − 0.596 to − 1.302), but not a statistically negative impact that is significant at a later stage (2011–2012). It is also important, that high education levels increased the chance of drinking alcohol when significant, being the coefficient for university graduated the most significant one. A further noteworthy point is that the impact of age turned negative among only-on-weekend drinkers, the estimation gets nearby − 0.04. Once again, statistically significant coefficients were found for male, married status and nationality, the first one being positive and the other two being negative.

**Table 3:** Multinomial Logit-model Estimation. Dependent Variable: *drinking- frequency*

	Base outcome = 0					
	(1)		(2)		(3)	
Variable						
Pre-crisis (2006)						
Unemployed	−1.302 (− 4.46)	***	− 0.665 (− 1.78)	*	− 0.596 (− 3.52)	***
Age	− 0.056 (− 6.33)	***	0.015 (1.31)		0.026 (4.07)	***
Male	1.267 (7.09)	***	1.527 (6.09)	***	1.353 (10.31)	***
Educ2	− 0.075 (− 0.29)		− 0.420 (−1.11)		−0.153 (− 0.81)	
Educ3	0.377 (1.61)		0.248 (0.80)		0.230 (1.30)	
Educ4	0.708 (2.22)	**	0.153 (0.33)		0.428 (1.67)	*
Educ5	0.884 (3.43)	***	0.973 (3.08)	***	0.635 (3.15)	***
Married	−0.540 (−2.97)	***	−0.054 (− 0.22)		− 0.033 (− 0.24)	
Nationality	− 0.388 (−1.20)		−0.205 (− 0.44)		−0.053 (− 0.21)	
Crisis (2011–2012)						
Unemployed	−0.079 (− 0.85)		− 0.695 (− 0.63)		−0.015 (− 0.23)	
Age	−0.030 (−9.16)	***	0.058 (1.72)	*	0.044 (19.62)	***
Male	1.557 (20.36)	***	2.702 (2.46)	***	1.821 (35.41)	***
Educ2	−0.418 (−4.57)	***	−0.303 (− 0.30)		− 0.126 (−1.96)	**
Educ3	1.032 (6.92)	**	0.405 (0.32)		0.445 (5.52)	***
Educ4	1.023 (5.81)	***	1.805 (1.43)		0.495 (4.47)	***
Educ5	1.201 (7.81)	***	−12.119 (−0.03)		0.655 (7.52)	***
Married	−0.376 (−4.80)	***	−1.425 (− 1.66)	*	− 0.069 (− 1.34)	
Nationality	− 0.501 (− 4.05)	***	2.735 (3.37)	***	− 0.346 (− 3.91)	***

*Drinking-frequency*: 0 if does not drink alcohol, 1 if drinks alcohol only on weekends, 2 if drinks alcohol only on weekdays, and 3 if drinks alcohol on weekends and weekdays alike

*Education* is categorized in terms of five levels of educational attainment (*Educ1*-*Educ5*, with *Educ1* “primary education or below” being the reference category *Educ2*: Compulsory secondary education; *Educ3*: Non-compulsory and pre-university secondary education; *Educ4*: Specific labour training; *Educ5*: University graduate

*z*-statistics in parentheses

Observations: 4079 for 2006 and 11,321 for 2011–2012

Reference category: the reverse one for each dichotomous variable

***p < 0.01, **p < 0.05, *p < 0.10.

Notwithstanding, an analysis of matching-techniques that provides comparisons of different points in the transition along the business cycle is conducted. Table 4 shows alternative ATT estimates from Eq. (4) for “unemployed” (column 1), the counterfactual as if they were employed (column 2), and the difference between the previous two (column 3). Furthermore, as can be also seen in this table, drinking patterns through the recession significantly differed from the boom period. Column 3 indicates that the ATT (impact) from “nearest neighbor without replacement” ranges from − 0.033 in 2006 to 0.013 in period 2011–2012. There is clear evidence here that being unemployed prior to the crisis did not raise the incidence of heavy alcohol drinking. Conversely, it seems to have had a positive association in the crisis. Hence, the observed sizeable excess indicates a positive though non-significant effect on heavy alcohol drinking. These findings are robust to alternative matching methods.

**Table 4:** Impact Estimates of Unemployment on Drinking Behavior 2006 and 2011–2012. Dependent Variable: *drinker*

	Unemployed E(Y_1_|D = 1)	Counterfactual E(Y_0_|D = 1)	ATT (Impact)	
Pre-crisis (2006)				
Nearest neighbour with replacement	0.197	0.228	−0.031 (−1.37)	
Nearest neighbour without replacement	0.197	0.229	−0.033 (− 3.37)	***
Nearest neighbour with more than one neighbour (3)	0.197	0.217	−0.021 (−1.38)	
Radius matching (0.02)	0.197	0.227	−0.030 (− 2.94)	***
Kernel	0.197	0.237	−0.047 (−3.90)	***
Crisis (2011–2012)				
Nearest neighbour with replacement	0.254	0.246	−0.008 (− 0.37)	
Nearest neighbour without replacement	0.254	0.241	0.013 (1.46)	
Nearest neighbour with more than one neighbour (3)	0.254	0.242	0.012 (0.86)	
Radius matching (0.02)	0.254	0.251	0.003 (0.26)	
Kernel	0.254	0.252	0.002 (0.20)	

Full model that adjusts for age, gender, education, marital status, and nationality in each year. Average treatment effect on the treated (ATT) is the difference between the two previous columns. *t*-statistics in parentheses

***p < 0.01, **p < 0.05, *p < 0.10

Last, we consider how business cycles affect drinking patterns. To this end, we estimate Eq. (5) and report the results in Table 5. In row 1, a drop in the incidence of drinking behavior is observed (δ = − 0.150), i.e., lower drinking levels in the crisis than previously. Besides, row 2 presents the estimates of drinking for the unemployed individuals. The coefficient of γ = − 0.214 is significantly negative, that is the unemployed would drink less than the employed. The absence from drinking is consistent with both the uncovering hypothesis (it predicts a reduction in the frequency and intensity of alcohol drinking among people of working age, by the threat of job loss if they continue to drink) and the income-effect hypothesis (foresees reductions in alcohol use throughout the population, but particularly among lower-income people who would be the most sensitive to income losses). Then, estimates in row 3 present a significantly positive interaction-term (ϕ = 0.234). This finding suggests that being an unemployed relatively increased drinking in the crisis related to the pre-crisis; that is, economic downturn contributed to explaining heavy alcohol drinking variations of the unemployed. These results provide further evidence, both that the unemployed usually drink less alcohol than the employed, and in the crisis they comparatively did so more than in the boom. Along with estimates from Table 3, there are significant drinking differences in 2006 between the unemployed and the employed. However, this pattern is no longer significant in period 2011–2012. Estimates from the alternative specifications are reported in the Additional file 1: Tables A.1-A.3. The first analysis addresses potential concerns about the exogeneity of the explanatory variables. The main warning comes from potentially unmeasured factors that may affect drinking behaviors and correlate with unemployment status. This issue is investigated by pooling surveys and adding time-varying controls into primary specifications.

**Table 5:** Estimates of drinking behavior from unemployment changes before and during the crisis

Effect	Full model with controls		Pseudo R^2^	*n*
Change in drinking behavior (δ)	−0.150	***	0.098	29,677
	(−4.46)			
Effect of unemployment on drinking (*γ*)	−0.214	***		
	(−3.19)			
Change in the effect of unemployment on drinking behavior during the crisis (θ)	0.234	***		
	(2.76)			

Full model that adjusts for age, gender, education, marital status, and nationality

*z*-statistics in parentheses

***p < 0.01, **p < 0.05, *p < 0.10

## Discussion

Our results support previous research indicating that alcohol drinking is a pro-cyclical phenomenon [20]. Accordingly and along with Ettner [21], alcohol drinking should be positively related to real disposable income (with respect to wages) and benefits from family support or insurance mechanisms [22]. When people actually realize that hard economic times are here to stay, they then apply a regime of austerity. Since alcoholic beverages are among the not indispensable items in consumer goods, they could no longer find a place in the household budget.

Our evidence also supports that alcohol drinking is largely a function of socioeconomic characteristics. More specifically, dual influences of education on alcohol abuse through the awareness of health capital and via income are expected. Similarly, family responsibilities would limit the participation in festivities and the risk of drinking alcohol to excess [13]. This attitude would be also being expected from foreigners. It should be taken with caution because this group appears to be small and is likely to be quite heterogeneous. Besides, estimates of the age associations with drinking are consistent with the findings of Aghion, Howitt, and Murtin [23]. Accordingly, young individuals should be made more aware than older individuals about the fact that health capital might well be preserved in order to enhance their future well-being.

Generally, our findings allow policymakers to develop better intervention programs for improving health. Public policy design for alcohol consumers would require both prevention and recovery strategies to be met as goal towards constructing health and labor pillars. In light of the implications that can be drawn from the results of the empirical study, they may be helpful in driving interventions programs towards reducing alcohol consumption in population segments where it is more frequent, echoing earlier findings. The unemployed people usually drink more during recession, so the unemployed may warrant special intervention efforts. This is because new measures are required to recover competitiveness and to strengthen employment, by focusing on training and the updating of workers’ skills. In addition, it is worth highlighting that chronic drug consumption (i.e., problem drinking with respect to alcohol) reduces the probability of being employed [12]. Therefore, tackling socio-economic determinants is much more likely to be effective at addressing alcohol-related harms in the population. Better education may also lead to social situations where alcohol is consumed. Should this be the case, it may be desirable to design intervention programs that are driven to involve those people who have a higher level of education. Likewise, stratified analysis revealed distinct alcohol consumption patterns across gender subgroups. Since females are more conscious of the harmful effects of alcohol consumption than males, governments can further curtail alcohol consumption by focusing their efforts on males [24].

But it is not the full picture since we should highlight the research limitations of the study. First, we refer to correlation between variables, not causation (that would require that changes in one variable would directly cause changes in the other one). It is important that one does not commit the ecological fallacy in the interpretation of statistical data [25]. Here, it would be to assume that aggregate unemployment correlations will translate into individual correlations. Second, the 2006 survey responders are not the same as the 2011–2012 responders. Furthermore, we are limited by the variables included within the National Health Survey(s), in future rounds of the SNHS authors claimed the importance of connection (how questions are formulated between surveys. Besides, if possible, to include more questions regarding these lifestyles topics). By focusing on alcohol-use, we are not trying to divert attention away from other major recovery challenges. But, rather we aim at enhancing health capital which can stimulate economic growth and citizens’ well-being, thereby reducing unemployment and social inequality. Future directions for the study, when more data would be available, should contemplate analysis by age groups. Furthermore, a special consideration to social class would be really interesting.

All in all, more studies are needed to examine socioeconomic inequalities and the impact of the economic recession on health outcomes [26]. Consistent with this, if policymakers do not account for the alcohol consumption link that is related with the unemployed, then strategies will surely be misguided.

## Conclusions

This paper contains new empirical evidence on drinking behavior over the business cycle. The episode analyzed was the 2008 economic collapse in Spain with its long-lasting effects, whereby high unemployment rates were included. We found that being unemployed prior to the crisis reduced the chance of alcohol drinking with regard to the employed. However, differences tended to vanish following the economic downturn along with lower overall alcoholic beverage consumption. Additionally, the present study showed that changes in alcohol consumption and drinking patterns were related to the characteristics of individuals. Hence, there were mixed reactions to the shock, depending on the education level and the gender, among other features.

## Additional file

Additional file 1: **Table A.1.** Logistic Estimation from the Pooled Surveys. Dependent Variable: *drinker.* **Table A.2.** Multinomial Logit-model Estimation from the Pooled Surveys. Dependent Variable: *drinking-frequency.* **Table A.3.** Estimates of Drinking from Unemployment Changes Before and During the Crisis by Gender. (DOCX 38 kb)

## Abbreviations

ATT: average treatment effect on the treated
INE: Spanish National Institute of Statistics
SNHS: Spanish National Health Survey
