# Supplementary material for: Do the unemployed hit the bottle during economic downturns? An empirical approach for Spain
Source: BMC Public Health. 2019 May 7;19:523. doi: 10.1186/s12889-019-6882-2 (PMC6505297; doi:10.1186/s12889-019-6882-2)
Supplement: Supplementary file 1 — Table A.1. Logistic Estimation from the Pooled Surveys. Dependent Variable: drinker. Table A.2. Multinomial Logit-model Estimation from the Pooled Surveys. Dependent Variable: drinking-frequency. Table A.3. Estimates of Drinking from Unemployment Changes Before and During the Crisis by Gender. (DOCX 38 kb) [file 12889_2019_6882_MOESM1_ESM.docx]

**Additional file 1**

**Table A.1** Logistic Estimation from the Pooled Surveys. Dependent Variable: *drinker*

| Variable/ specification | (1) | | (2) | | (3) | | (4) | | (5) | | (6) | |
| --- | --- | --- | --- | --- | --- | --- | --- | --- | --- | --- | --- | --- |
|  | ^Odds ratio^ | ^95% CI^ | ^Odds ratio^ | ^95% CI^ | ^Odds ratio^ | ^95% CI^ | ^Odds ratio^ | ^95% CI^ | ^Odds ratio^ | ^95% CI^ | ^Odds ratio^ | ^95% CI^ |
| Unemployed | 0.931^*^ | [0.859-1.010] |  |  |  |  |  |  |  |  |  |  |
| Unem_never |  |  | 0.654^*^ | [0.414-1.034] |  |  |  |  |  |  |  |  |
| Unem_6 |  |  |  |  | 1.084 | [0.955-1.232] |  |  |  |  | 1.069 | [0.941-1.214] |
| Unem_6_12 |  |  |  |  |  |  | 0.852 | [0.699-1.040] |  |  | 0.847^*^ | [0.694-1.034] |
| Unem_12 |  |  |  |  |  |  |  |  | 0.872^**^ | [0.776-0.979] | 0.871^**^ | [0.776-0.979] |
| Age | 1.030^***^ | [1.027-1.032] | 1.030^***^ | [1.027-1.032] | 1.030^***^ | [1.027-1.033] | 1.030^***^ | [1.027-1.032] | 1.030^***^ | [1.027-1.033] | 1.030^***^ | [1.027-1.033] |
| Male | 4.339^***^ | [4.093-4.600] | 4.338^***^ | [4.092-4.598] | 4.345^***^ | [4.099-4.606] | 4.343^***^ | [4.098-4.605] | 4.340^***^ | [4.094-4.601] | 4.340^***^ | [4.094-4.601] |
| Educ2 | 0.995 | [0.926-1.069] | 0.993 | [0.923-1.066] | 0.991 | [0.922-1.065] | 0.994 | [0.925-1.068] | 0.995 | [0.926-1.068] | 0.995 | [0.926-1.069] |
| Educ3 | 1.010 | [0.939-1.087] | 1.011 | [0.939-1.088] | 1.014 | [0.942-1.091] | 1.012 | [0.941-1.090] | 1.011 | [0.939-1.088] | 1.011 | [0.939-1.088] |
| Educ4 | 1.059 | [0.948-1.183] | 1.061 | [0.951-1.186] | 1.067 | [0.955-1.191] | 1.064 | [0.953-1.189] | 1.059 | [0.949-1.183] | 1.061 | [0.950-1.184] |
| Educ5 | 0.994 | [0.914-1.081] | 0.998 | [0.918-1.086] | 1.002 | [0.922-1.090] | 0.999 | [0.918-1.086] | 0.994 | [0.914-1.081] | 0.994 | [0.914-1.081] |
| Married | 0.898^***^ | [0.846-0.952] | 0.900^***^ | [0.849-0.954] | 0.902^***^ | [0.851-0.956] | 0.900^***^ | [0.849-0.954] | 0.898^***^ | [0.846-0.951] | 0.897^***^ | [0.846-0.951] |
| Nationality | 0.697^***^ | [0.629-0.773] | 0.695^***^ | [0.627-0.771] | 0.694^***^ | [0.626-0.769] | 0.696^***^ | [0.627-0.772] | 0.696^***^ | [0.628-0.772] | 0.696^***^ | [0.628-0.772] |
| t | 0.886^***^ | [0.832-0.943] | 0.883^***^ | [0.830-0.940] | 0.881^***^ | [0.828-0.938] | 0.883^***^ | [0.830-0.940] | 0.887^***^ | [0.834-0.945] | 0.889^***^ | [0.834-0.946] |
| Pseudo R^2^ | 0.010 | | 0.010 | | 0.010 | | 0.010 | | 0.010 | | 0.098 | |

Observations: 29,677.

Reference category: the reverse one for each dichotomous variable.

*** p < 0.01, ** p < 0.05, * p < 0.10

**Table A.2** Multinomial Logit-model Estimation from the Pooled Surveys. Dependent Variable: *drinking-frequency*

| Variable | Base outcome = 0 | | | | | | |
| --- | --- | --- | --- | --- | --- | --- | --- |
|  | (1) | | (2) | | (3) | | |
| Unemployed | -0.213 | ^**^ | -0.150 |  | -0.049 |  | |
|  | (-2.44) |  | (-0.45) |  | (-0.82) |  | |
| Age | -0.033 | ^***^ | 0.032 | ^***^ | 0.043 | ^***^ | |
|  | (-11.13) |  | (3.47) |  | (20.78) |  | |
| Male | 1.574 | ^***^ | 1.908 | ^***^ | 1.757 | ^***^ | |
|  | (23.10) |  | (8.73) |  | (37.81) |  | |
| Educ2 | -0.279 | ^***^ | -0.407 |  | -0.135 | ^**^ | |
|  | (-3.42) |  | (-1.29) |  | (-2.39) |  | |
| Educ3 | 0.758 | ^***^ | 0.481 | ^*^ | 0.465 | ^***^ | |
|  | (7.29) |  | (1.88) |  | (6.92) |  | |
| Educ4 | 0.833 | ^***^ | 0.434 |  | 0.518 | ^***^ | |
|  | (6.17) |  | (1.16) |  | (5.33) |  | |
| Educ5 | 1.007 | ^***^ | 0.965 | ^***^ | 0.681 | ^***^ | |
|  | (8.84) |  | (3.73) |  | (8.98) |  | |
| Married | -0.414 | ^***^ | -0.131 |  | -0.051 |  | |
|  | (-5.95) |  | (-0.65) |  | (-1.09) |  | |
| Nationality | -0.500 | ^***^ | 0.030 |  | -0.287 | ^***^ | |
|  | (-4.48) |  | (0.09) |  | (-3.60) |  | |
| t | -2.397 | ^***^ | -6.317 | ^***^ | -3.758 | | ^***^ |
|  | (-24.45) |  | (-15.30) |  | (-49.37) |  | |

*Drinking-frequency*: 0 if does not drink alcohol, 1 if drinks alcohol only on weekends, 2 if drinks alcohol only on weekdays, and 3 if drinks alcohol on weekends and weekdays alike.

*z*-statistics in parentheses.

Observations: 15,400. Reference category: the reverse one for each dichotomous variable.

*** p < 0.01, ** p < 0.05, * p < 0.10

**Table A.3** Estimates of Drinking from Unemployment Changes Before and During the Crisis by Gender

| Effect (males) | Full model  with controls | | Pseudo  R^2^ | *n* |
| --- | --- | --- | --- | --- |
| Change in drinking behavior ($\delta)$ | -0.107 | ^**^ | 0.028 | 14,335 |
|  | (-2.56) |  |  |  |
| Effect of unemployment on drinking $(\gamma$) | -0.237 | ^***^ |  |  |
|  | (-2.61) |  |  |  |
| Change in the effect of unemployment on drinking behavior during the crisis $\left( \theta\right)$ | 0.232 | ^**^ |  |  |
|  | (2.11) |  |  |  |

| Effect (females) | Full model  with controls | | Pseudo  R^2^ | *n* |
| --- | --- | --- | --- | --- |
| Change in drinking behavior ($\delta)$ | -0.206 | ^***^ | 0.017 | 15,342 |
|  | (-3.64) |  |  |  |
| Effect of unemployment on drinking $(\gamma$) | -0.178 | ^*^ |  |  |
|  | (-1.78) |  |  |  |
| Change in the effect of unemployment on drinking behavior during the crisis $\left( \theta\right)$ | 0.209 |  |  |  |
|  | (1.51) |  |  |  |

Full models that adjust for age, education, marital status, and nationality.

*z*-statistics in parentheses.

*** p < 0.01, ** p < 0.05, * p < 0.10
